# Comparison of digital protocols for the measurement of peri-implant marginal bone loss

**DOI:** 10.4317/jced.55396

**Published:** 2018-12-01

**Authors:** David Peñarrocha-Oltra, Ivan Palau, Guillermo Cabanes, Beatriz Tarazona, Maria Peñarrocha-Diago

**Affiliations:** 1 DDS, PhD. Assistant Professor of Oral Surgery and Implantology. University of Valencia Medical and Dental School; 2 DDS. University of Valencia Medical and Dental School; 3 Collaborating Professor of Oral Surgery. University of Valencia Medical and Dental School. Valencia, Spain; 4 DDS, PhD. Assistant Professor of Orthodontics. University of Valencia Medical and Dental School; 5 Full Professor of Oral Surgery. Professor of the Master in Oral Surgery and Implantology. University of Valencia Medical and Dental School. Valencia, Spain

## Abstract

**Background:**

The measurement of peri-implant marginal bone loss is currently carried out using digital methods of radiographic analysis assisted by various types of software. The purpose of this study was to compare the characteristics of three different softwares: specific radiology software for the development and visualization of radiological images in DICOM format (3Dicom Viewer®), advanced level software for professional editing of bitmap images (or raster graphics) (Adobe Photoshop®), and mid-level software for processing bitmap-type images, programmed in Java and in the public domain (ImageJ®).

**Material and Methods:**

It was verified that the three softwares used are valid for the measurement of peri-implant marginal bone loss provided that the appropriate protocol is fulfilled.

**Results:**

The results showed no significant differences between Adobe Photoshop® and ImageJ® with respect to 3Dicom Viewer® in the measurements of mesial and distal bone loss of the implants, without influence of the dental sector where they were located.

**Conclusions:**

The measurements made with ImageJ® looked more like those of the control software (3Dicom Viewer®) than those of Adobe Photoshop®, but with a greater degree of dispersion. Thus, Adobe Photoshop® is a slightly inaccurate method but with less dispersion.

** Key words:**Digital measurement, measurement software, peri-implant marginal bone loss, implants.

## Introduction

The quantity and quality of bone surrounding the implant is one of the essential factors for the medium and long-term success of this therapy and is decisive in the morphology, quality and aesthetics of soft tissue sealing in the implant-supported restoration (1). The radiological techniques indicated for peri-implant diagnosis are: periapical radiography, extraoral panoramic radiography and computerized tomography (conventional or cone-beam) (2). According to several studies, digital periapical radiography is the most indicated to assess the level of the bone crest. However, since it is a two-dimensional image, it is evident that in the case of vestibular and lingual bony defects, there are limitations of visualization (3).

The analysis of peri-implant bone loss has been well studied over the years. Traditionally, a “classic protocol” has been used in periapical radiography where two visible and easily identifiable reference points were located at each end, mesial and distal, of the implant platform. Several authors (4-8) modified this measurement protocol because the placement of implants in a subcrestal position implied an initial bone level above the implant platform. In this way, the authors assigned positive values when the bone was above the platform, value 0 when it was at the level of the platform and negative value when it was below. The current trend is the measurement through the use of specific software development and visualization of radiographs. The digital image that is obtained is composed of pixels (or bitmap), each of which is assigned a numerical value of position in the image and of luminosity in gray scale. In this way, it is possible to form the radiological image in the computer. Digital image software programs, in general, offer many tools for the analysis of these (9).

The aim of this study was to evaluate the validity of three different software: Specific radiology software for developing and displaying images in DICOM format (3Dicom Viewer®), advanced level software for professional editing of bitmap images (or graphics) rasterized) (Adobe Photoshop®), and free software, of medium level, for processing images of bitmap type, programmed in Java (ImageJ®).

## Material and Methods

The study protocol was approved by the Ethics Committee for Research Involving Human Subjects at the University of Valencia, Spain (H1506593103796). Rights have been protected by the Institutional Review Board. All subjects gave their informed consent to take part in the study. Any data that might disclose the identity of the subjects under study have been omitted. This study was designed following the Helsinki declaration and the STROBE statement (10).

150 dental implant x-rays taken at the Master of Oral Surgery and Implantology at the University of Valencia were selected. The sample of implants studied was selected based on the inclusion and exclusion criteria. The inclusion criteria applied were well-parallel digital radiographs: 1) initial on the day of implant placement; 2) follow-up from 2 to 5 years. The exclusion criteria applied were: 1) less than 2 years of follow-up; 2) badly parallel radiographs; 3) X-rays with the presence of artifacts that prevent measurement. All the implants were from the Ticare® commercial house.

To assess the level of bone with respect to the coronal part of the implant, digital intraoral plates of adults with a size 2 of 31x44 millimeters were used as a radiographic film. These radiographs were obtained with a radiology unit (Novelix 708 CCX, Trophy, Marne-la-Vallée, France) using the parallelization method with a Rinn XCP ring positioner (Dentsply, Constanz, Germany), allowing parallelization between the tube X-rays and the movie.

In the measurement of peri-implant bone loss, the three previously mentioned software were used: 3Dicom Viewer® radiographic vision software (3Dicom Viewer®, 3Dicom, Castellón, Spain), Adobe Photoshop® bit image editing software (Adobe Photoshop®, Adobe, San Jose, CA, US) and ImageJ® image processing software (ImageJ®, National Institute of Mental Health, Bethesda, MD, US).

-Methodology

**Figure 1:**
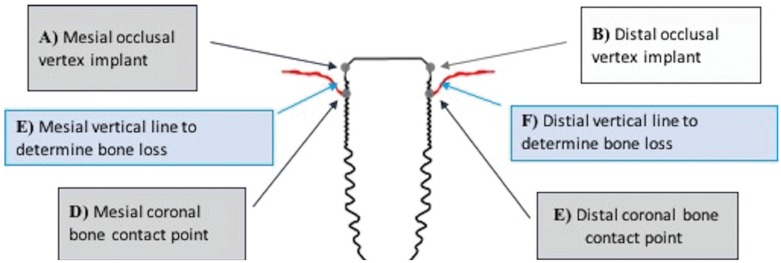
Assessment of bone loss from reference points.

In order to make measurements of bone loss around the implants, four stable reference points were determined in the radiological image. Two reference points were established within the head of the implant, one mesial (A) and one distal (B). These points coincided with the vertex of the occlusal table of the implant. On the other hand, two other reference points (C and D) were established, which coincided with the most coronal bone contact in mesial and distal (Fig. 1).

To determine the bone loss on both sides of the implant (E and F), the first reference point (A and B) was joined with the second (C and D) by a line, which was quantified in units of length of the metric system international (centimeters and millimeters, depending on the software applied) (Fig. 1).

The entire sample was analyzed by the three softwares:

• 3Dicom Viewer®: digital protocol of the radiological vision software.

• Adobe Photoshop®: digital protocol of the image editing software.

• ImageJ®: digital protocol of the image processing software.

-Data analysis

The measurement with 3Dicom Viewer® was considered as gold-standard and, therefore, the comparison of each of the techniques with it will allow to conclude about its validity. The absolute difference between the measurement of peri-implant bone loss in the digital protocols of Adobe Photoshop® and ImageJ® and 3Dicom Viewer® was computed, both mesially and distally. In addition, the adjustment to normal distribution of the differences was verified by the Kolmogorov-Smirnov test.

On the other hand, the mean values of the measurements obtained by a technique were compared with 3Dicom Viewer®, by means of a paired measures test. This allowed concluding on the absence of bias, a necessary condition to ensure the validity of each study group.

To study the agreement between the study method and the control method, a simple linear regression model was estimated, obtaining confidence intervals for the coefficients. If the interval of the constant contained 0 and that of the slope to 1, it could be accepted that the main diagonal or bisector is the ideal adjustment line and, consequently, the test method would be valid. Also, Pearson’s linear correlation coefficient was provided.

Finally, in order to explore whether the degree of precision of a technique depends on the position of the implant, on which the bone loss is measured, nonparametric tests were applied Mann-Whitney and Kruskal-Wallis respectively for the analysis by sector (3 groups: Incisors / Canines, Premolars, Molars). The statistical analysis was carried out by DP.

## Results

The final sample of the study was constituted by 134 implants, of which 60 were placed in the maxilla and 74 in the mandible. 28 of the implants were placed in the incisal area, 48 in the premolar area and 58 in the molar area. The implants were placed in 134 patients (72 men and 62 women) with an age range of 44.2 years (range of 21 to 62 years).

-Reproducibility

 Table 1 shows the basic descriptive statistics of the measurements of mesial and distal bone loss with the different techniques. An average mesial loss of 0.66 ± 0.94 mm is observed for 3Dicom Viewer®, 0.64 ± 0.89 mm for Adobe Photoshop® and 0.71 ± 1 mm for ImageJ®. In distal 3Dicom Viewer® observed an average bone loss of 0.8 ± 1.1 millimeters, 0.72 ± 1.04 millimeters in Adobe Photoshop® and 0.78 ± 1.1 millimeters in ImageJ®.

**Table 1:**
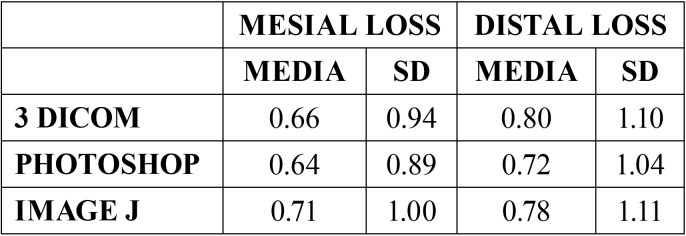
Mean and standard deviation (SD) of mesial and distal bone loss according to the technique used (millimeters).

On the other hand,  Table 2 shows the average values of the differences of each study method and the control method, as well as the values relative to the error of the method. In mesial, Adobe Photoshop® measures, on average, 0.02 mm less bone loss compared to 3Dicom Viewer®, while ImageJ® measures 0.05 more on average. In distal, Adobe Photoshop® measures, on average, 0.08 mm less bone loss compared to 3Dicom Viewer®, while ImageJ® measures 0.02 less on average.

**Table 2:**
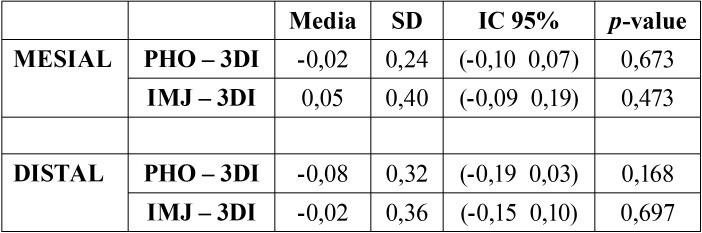
Difference in bone loss measurements between study methods and control method: mean ± standard deviation, 95% confidence interval and t-student test (p-value). (PHO=Adobe Photoshop®; 3DI= 3Dicom Viewer®; IMJ= ImageJ®).

 Table 3 shows that the confidence intervals for the constant and the slope are wider than in the case of Adobe Photoshop®, consistent with the greater dispersion observed. On the other hand, the slope coefficient is very close to 1, which means that the measurement does not deviate to one side as larger or smaller losses are measured. In short, the measurements with ImageJ® are globally more similar than those of Adobe Photoshop® to those of 3Dicom Viewer®, but with a greater degree of uncertainty.

**Table 3:**
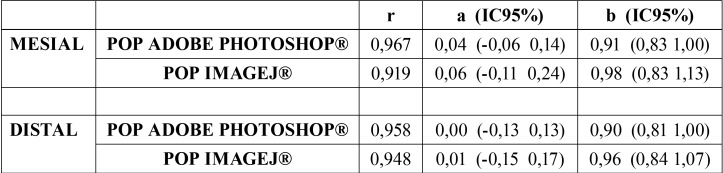
Concordance between measurements of the techniques. Results linear regression with independent variable POP (peri-implant bone loss) by 3Dicom Viewer®: Pearson r coefficient, coefficients of the constant equation (a) and slope (b) and 95% confidence intervals (r = Pearson r coefficient; a = coefficients of the constant equation; b = coefficients of the slope equation; IC = confidence intervals).

**Figure 2:**
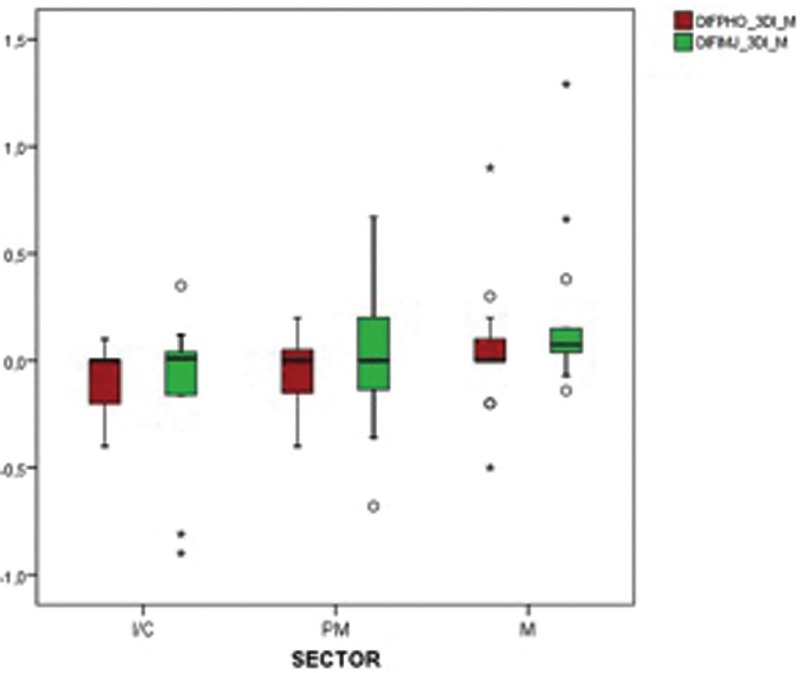
Differences in mesial peri-implant bone loss PHO-3DI and IMJ-3DI in the different groups of teeth. (Abbreviations: DIF = Mean differences and confidence intervals, PHO = Photoshop, 3DI = 3Dicom Viewer, IMJ = ImageJ, M = mesial ; D = distal).

Figure 2 shows, for mesial, the distribution of the differences of peri-implant bone loss between Adobe Photoshop® and 3Dicom Viewer® and between ImageJ® and 3Dicom Viewer® in the different groups of teeth. A Kruskal-Wallis test confirms that Adobe Photoshop® is equally valid whether it measures losses in the previous sector, as in Premolars or Molars (*p* = 0.449). The same can be applied to ImageJ®; although the tendency is to deviate more in the molars (*p* = 0.131).

**Figure 3:**
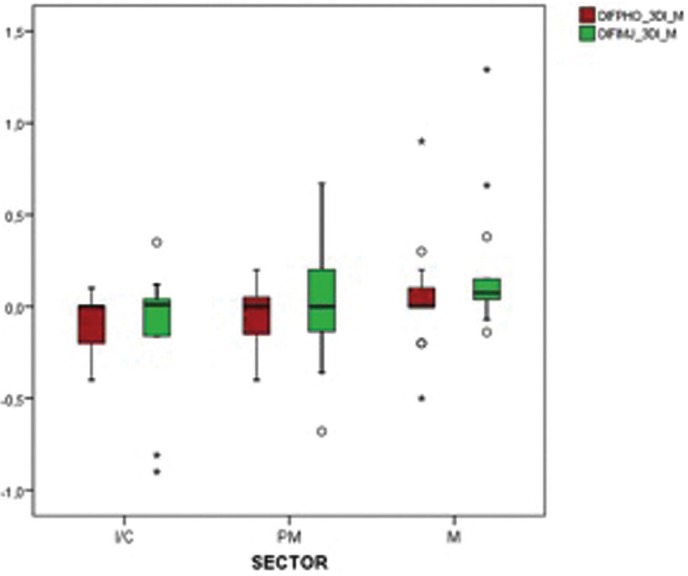
Differences in mesial peri-implant bone loss PHO-3DI and IMJ-3DI in the different tooth groups. (Abbreviations: DIF = Mean differences and confidence intervals, PHO = Photoshop, 3DI = 3Dicom Viewer, IMJ = ImageJ, M = mesial ; D = distal).

Of the same, in Figure 3 it is observed that for distal the same thing happens. No differences were found between positions neither for Adobe Photoshop® (*p* = 0.981, KW), nor for ImageJ® (*p* = 0.531).

## Discussion

The aim of this study was to evaluate the validity of three different software: Specific radiology software for developing and displaying images in DICOM format (3Dicom Viewer®), advanced level software for professional editing of bitmap images (or graphics) rasterized) (Adobe Photoshop®), and free software, of medium level, for processing images of bitmap type, programmed in Java (ImageJ®).

In the present study, mean values of peri-implant bone loss of 0.5 mm in mesial and 0.2 mm in distal were obtained. According to the success criteria of the implants established by Albrektsson (11), this marginal loss of bone would be a criterion of success in the implants analyzed, since the loss of 1.5 mm in the first year and vertical bone loss less than 0.2 mm per year after the first year since its placement. However, at the extremes of the recorded values, bone losses of 3.9 mm in mesial and 4.00 mm in distal were found, which would suppose a pathological bone loss according to the previously established criteria.

No studies have been found in the literature where this specific software is used. However, there are numerous studies where very similar software is used, specific to radiographic vision, for the assessment of peri-implant marginal bone loss, as is the case of Szymańska *et al.* (12), and Dave *et al.* (13), who use the Planmeca Romexis® software to evaluate the bone loss of the implants according to the neck of these, in the case of the first author, and to compare the diagnosis of peri-implant marginal bone loss with CBCT and with digital periapicals in the case of the second author. In the same sense, there are other authors who also evaluated this bone loss with other softwares of the same group, such as Cassetta *et al.* (7,14), with the VixWinPRO® program, Peñarrocha *et al.* (15), with Digora Optime®, or Van Weehaeghe *et al.* (16), with the Mediadent® program.

Adobe Photoshop®, certain advantages and disadvantages with respect to the 3Dicom Viewer® control software. Among the positive points of this computer program, we found that it allowed the application of yellow filters, to favor the differentiation of radiographic densities, the use of “horizontal guide lines” that facilitated the location of the measurement level at its initial and final points, as well as the layer overlay tool, as explained in the study by Fernández *et al.* (17). Based on this software, several authors have been found in the literature who have used it to perform their measurements of bone loss in implants. Koutouzis *et al.* (18), used it to retrospectively analyze the potential influence of implant tilt on marginal bone loss in fixed partial dentures supported by implants during a 5-year functional loading period. Nisapakultorn *et al.* (19), they used it to evaluate the factors that affected marginal bone loss in implants. Finally, Gheisari *et al.* (20), they used it to evaluate the bone loss of the implants placed in a step and those placed in two surgical steps.

Regarding the advantages and disadvantages of the ImageJ® digital protocol, as shown in the published literature (21,22,23), it is an adequate software to perform measurements in implantology, since it is designed specifically for perform measurements in medicine. It is free and open access software, so any user can use it easily. Also, within its advantages, we can include that it offered its results in millimeters and saved them in a history of measurements, being able to consult at all times the values obtained previously. As a drawback, it also required a previous calibration of the image, although through a faster, simpler and more intuitive procedure than in Photoshop®. In addition, it presented a greater learning curve with respect to 3Dicom Viewer®, which was another disadvantage of the software. In addition, it presented a greater learning curve with respect to 3Dicom Viewer®, which was another disadvantage of the software. It should be noted that numerous studies have been found in the literature that used it to measure peri-implant marginal bone loss. Romeo *et al.* (22,23), in addition to indicating it as the appropriate computer program to measure bone loss in implants, they use it to evaluate the medium and long-term prognosis of cantilever fixed prostheses on implants and to evaluate the clinical effectiveness of implants. different sizes in different bony areas of the host. Also, Sivolella *et al.* (24), they used it to investigate the medium and long-term prognosis of short implants in edentulous edentulous patients. On the other hand, Mendoza *et al.* (9), they used it to compare the marginal alteration of the bone level through radiographic evaluation in short and standard implants. In contrast, Francesco *et al.* (25), evaluated with him, through a clinical and radiographic analysis, the peri-implant bone resorption of tantalum dental implants. Finally, Dias *et al.* (21), based on the protocols designed and used by Romeo et al., It is used to evaluate bone healing and evaluate the position of Bone contact.

Finally, analyzing the statistical values of each software it is observed that both Adobe Photoshop® and ImageJ® are valid for the measurement of peri-implant marginal bone loss, since they do not present statistically significant differences with 3Dicom Viewer® (*p* = 0.673 mesial and *p* = 0.168 distal for Adobe Photoshop®, *p* = 0.473 mesial and *p* = 0.697 distal for ImageJ®). There were no differences in the analytical study by dental sectors (*p* = 0.499 for anterior and posterior sectors for Adobe Photoshop®, and *p* = 0.131 for ImageJ®). Likewise, no significant differences were found when measuring between the dental arches (*p* = 0.981 between arches for Adobe Photoshop® and *p* = 0.531 for ImageJ®). Therefore, Adobe Photoshop® and ImageJ® were valid for measurements of bone loss in implants no matter where they were placed. However, some nuances can be highlighted. On the one hand, analyzing Adobe Photoshop® software shows that it is a slightly inaccurate method, although it is related to the measurement of bone loss, it tends to underestimate the true values obtained by 3Dicom Viewer®. On the other hand, ImageJ® is a more imprecise imprecise method (more dispersion of results), but more accurate. That is to say, its regression line literally overlaps that of perfect agreement, but it is easier to find large point deviations of the periimplant marginal marginal bone loss.

As for the limitations of the study, as it is a retrospective study, on radiographs There may have been variation at the moment of its realization, which may affect, in a certain way, the capacity of comparison between pairs of radiographs, likewise it makes impossible the use of the “layer overlay tool” of Adobe Photoshop ® (17).

## Conclusions

The three analyzed software (3Dicom Viewer®, Adobe Photoshop® and ImageJ®) are valid for peri-implant marginal bone measurement provided that the appropriate protocol is followed. No differences were detected in the degree of precision of each technique depending on the position of the implant involved. Adobe Photoshop® is a slightly inaccurate method, but more accurate (little dispersion in results). ImageJ® is a somewhat more imprecise method, but more accurate (measures more similar to the control method).
